# Cases Admitted under the Care of the Surgeon of the Finsbury Dispensary, St. John's Square, Clerkenwell, from the 10th of September to the 10th of October, 1802

**Published:** 1802-11-01

**Authors:** J. Ricards

**Affiliations:** 68, Harton Street


*Cases admitted under the Care of the Surgeon of the Finsbury Dispensary, St. John's Square, Clerkenwell, from the 10th of September to the 10th of October, 1802.*

| | | | |
|---|---|---|---|
| Ophthalmia | 1 | Combuſtura | 1 |
| Eryſipelas | 1 | Lues | 3 |
| Abſceſſus Artuum | 2 | Gonorrhœa | 1 |
| ——— Mammarum | 2 | Vacciola | 10 |
| ——— Cervicis | 1 | Scrophula | 3 |
| Ulcus Palati | 1 | Eruptiones Chronicæ | 3 |
| Ulcera Artuum | 15 | Tumor Genu | 1 |
| Fiſtula in Ano | 2 | Hernia | 1 |
| Leucoma | 1 | Prolapſus Ani | 1 |
| Fractura Claviculæ | 1 | Polypus Naſi | 1 |
| ——— Ulnæ | 1 | Calculus in Urethra | 1 |
| ——— Patellæ | 1 | | — |
| ——— Coſtæ | 1 | Total | 62 |
| Contuſiones | 6 | | |

The patient, in the above liſt, who applied for the relief of a leucoma, or opacity of the cornea, has for many years been blind of the right eye, in conſequence of a blow, which produced a complete cataract. About a year ago, he had the miſfortune to receive a ſmall portion of the bark of a tree, which he was peeling, into the left eye, which was productive of conſiderable inflammation; he uſed various methods to remove this extraneous body, which he thought he ſtill felt in his eye, and could likewiſe perceive a dark ſpot on looking into it; he applied alſo to a Surgeon, who waſhed his eye with injections, and made uſe of different means to afford him relief, but without effect, and indeed with aggravation of pain. When he applied to me, his eye was much inflamed, extremely irritable, and ſenſible of the ſlighteſt ſtimulus; and on examining it with attention, I perceived an opacity of the tranſparent cornea, at the lower part, near its circumference; the centre of this was completely opake, and from this centre iſſued radii of opacity, which extended to ſome diſtance, but poſſeſſed more tranſparency than the central point; which, added to the vaſcular diſtention, nearly deprived him of the ſight of this eye likewiſe. To the view, this part ſeemed to project conſiderably beyond the other parts of the cornea; I therefore made a ſlight puncture into the prominent part, when immediately was diſcharged a ſubſtance which, on examination, appeared to be of a ſoft, calcareous ſtructure, and in its centre, as a kind of nucleus, was contained a very ſmall, dark-coloured flake, of a ſomething which, in all probability, was the portion of bark that was the original aggreſſor, which had gained a lodgment between the lamina of the cornea, and on which the calcareous depoſit had taken place. After the removal of this ſubſtance, the inflammation mation fubfided; the furrounding opacity is gradually difappearing, and little remains except a flight excavation in the cornea, at the part from whence this extraneous fubftance had been taken.

The only circumftance, perhaps, in the above cafe, which is worthy of notice, is, that the fluids of the eye, as well as the faliva, bile, urine, &c. are capable of forming earthy depofitions, if they have a nucleus afforded to them on which fuch concretions can take place.

The difeafe Leucoma, or Albugo, is one which is very frequent, and very difficult and tedious in being cured. The difeafe appears, as far as I have obferved, to arife under two principal forms; either an opacity without any elevation, from an effufion of coagulable lymph between the lamina of the cornea, affuming the appearance of a diffufed white cloud; or, an opake fpot, of rather a darker appearance, and elevated above the furface of the cornea. Thefe latter are of different extents, and, I believe, moft commonly arife from fome fubftance wounding the eye, or from fome fmall particle of matter which may have entered the eye, and injured the furface of the cornea: whereas the former fpecies is a confequence of general ophthalmia very frequently. The former fpecies fometimes gradually fubfides by the fpontaneous action of the abforbents, after inflammation is removed; if not, our reliance muft be in promoting the action of thefe veffels, not only generally, by evacuants and mercury, but topically, by blifters near the parts, which fhould be kept open, to eftablifh a local drain; and the application of ftimulants to the eye itfelf. A Cafe which I have at prefent under my care, has been repeatedly yielding to this mode of treatment, but the eye in this patient is fo extremely irritable, that frefh inflammations are produced by any ftimulus to the part, fometimes even by a blifter to the temple, or by a change in the atmofphere from heat to cold. The other fpecies of leucoma, which appears fituated on the external furface of the cornea, may fometimes be removed by the knife, but not always, as there is frequently fo much diffufed opacity attending it, as to render fuch an operation impracticable. Thefe cafes fometimes yield to ftimulating applications to the eye itfelf, affifted by fuch general means as excite abforbent action; various are the applications which are recommended for the purpofe of deftroying thefe opake effufions; fome with an intention of acting mechanically, others merely proving ftimulant. The worft cafe of this kind which I ever knew cured, was in a woman who received the difeafe from a local injury, by which (it nearly extending over the whole lucid cornea) fhe was almoft blind. In this cafe I only ordered a few drops, daily, to be admitted into the eye, of aq. cupri ammon. by which, in an aftonifhingly fhort fpace of time, the opacity was entirely removed.

Several

Several months ago, the Medical and Phyſical Journal contained a Paper on the removal of the paroxyſm of Gout by abſtracting the ſuperabundant heat from the inflamed part by cold applications, as is uſual in other inflammations, in oppoſition to the cuſtomary but (in the opinion of the Author of that paper) uſeleſs, and even miſchievous practice of enveloping the diſeaſe in flannels, furs, and ſuch other coverings as prevent the tranſmiſſion of any of the heat generated in the part. I was informed, not long ſince, of a gentleman who generally ſucceeds in preventing the acceſſion of a fit of gout by plunging his feet into cold water on its firſt approach, and continually renewing the cold application for a conſiderable time. I have recently directed ſimilar treatment in a caſe of gout; which, although not ſtrictly chirurgical, I hope I ſhall be excuſed in mentioning. A woman who was a patient of mine at the Diſpenſary, in conſequence of a diſlocation of the wriſt, was laſt week attacked by a fit of gout in her feet; a diſeaſe to which ſhe had been a martyr for many years, and generally ſuffered for the ſpace of five or ſix weeks under each attack. I directed her to keep her feet conſtantly wet with a cold application, by means of linen, which was to be renewed and changed as often as the liquid ſhould acquire any degree of heat: The application gave immediate relief, and in a few days ſhe was able to walk nearly a mile to the Diſpenſary, although the attack was ſo ſevere as to confine her to bed when this treatment was begun.

I think it right to record caſes which in any reſpect differ in their appearances from what is uſual, or yield to treatment which differs from the cuſtomary practice; but it is very much the faſhion, at the preſent day, to pay too much attention, and to place too much reliance on ſolitary caſes. I have known practitioners, who in other reſpects were men of undoubted good ſenſe and ability, who have ſuffered their minds to be ſo much miſled by the favorable termination of one or two caſes, under particular treatment, as to have eſtabliſhed a creed in their own breaſts, which they have implicitly believed, that a ſimilar remedy will always have the ſame reſult, and have formed theories of diſeaſe upon ſo ſhallow a baſis. Every phenomenon which we have never obſerved before, every appearance in diſeaſe, or effect of remedy which is new to us, ſhould be carefully marked and recorded; but we ſhould go no further, till, by a repetition of ſimilar phenomena, appearances and effects, again and again, we are authoriſed in the belief that ſuch and ſuch circumſtances are immutable reſults and eſtabliſhed laws. A ſingle fact ſhould not influence our judgement; but in a collection of ſimilar facts, ariſing from ſimilar circumſtances, each one individually will prove valuable, as an addition to the maſs of evidence contained in the whole.

